# PCR Strategies for Complete Allele Calling in Multigene Families Using High-Throughput Sequencing Approaches

**DOI:** 10.1371/journal.pone.0157402

**Published:** 2016-06-13

**Authors:** Elena Marmesat, Laura Soriano, Camila J. Mazzoni, Simone Sommer, José A. Godoy

**Affiliations:** 1 Department of Integrative Ecology, Estación Biológica de Doñana (CSIC), Sevilla, Spain; 2 Berlin Center for Genomics in Biodiversity Research (BeGenDiv), Berlin, Germany; 3 Leibniz Institute for Zoo and Wildlife Research (IZW), Berlin, Germany; 4 Institute of Evolutionary Ecology and Conservation Genomics, University of Ulm, Ulm, Germany; King Abdullah University of Science and Technology, SAUDI ARABIA

## Abstract

The characterization of multigene families with high copy number variation is often approached through PCR amplification with highly degenerate primers to account for all expected variants flanking the region of interest. Such an approach often introduces PCR biases that result in an unbalanced representation of targets in high-throughput sequencing libraries that eventually results in incomplete detection of the targeted alleles. Here we confirm this result and propose two different amplification strategies to alleviate this problem. The first strategy (called pooled-PCRs) targets different subsets of alleles in multiple independent PCRs using different moderately degenerate primer pairs, whereas the second approach (called pooled-primers) uses a custom-made pool of non-degenerate primers in a single PCR. We compare their performance to the common use of a single PCR with highly degenerate primers using the MHC class I of the Iberian lynx as a model. We found both novel approaches to work similarly well and better than the conventional approach. They significantly scored more alleles per individual (11.33 ± 1.38 and 11.72 ± 0.89 vs 7.94 ± 1.95), yielded more complete allelic profiles (96.28 ± 8.46 and 99.50 ± 2.12 vs 63.76 ± 15.43), and revealed more alleles at a population level (13 vs 12). Finally, we could link each allele’s amplification efficiency with the primer-mismatches in its flanking sequences and show that ultra-deep coverage offered by high-throughput technologies does not fully compensate for such biases, especially as real alleles may reach lower coverage than artefacts. Adopting either of the proposed amplification methods provides the opportunity to attain more complete allelic profiles at lower coverages, improving confidence over the downstream analyses and subsequent applications.

## Introduction

Multigene families play a key role in many research fields such as immunogenetics [1,2], embryology [3,4], kinship recognition [5] mating preferences [6], and olfaction and taste perception [7,8]. The advent of high-throughput sequencing has paved the way towards the genetic characterization of such regions due to two of its features: i) parallel sequencing eliminates the need to physically separate the co-amplified molecules prior to sequencing, and ii) large sequencing output allows each sample to be sequenced at a high coverage. This makes the analysis of a high number of samples cost- and labour-effective in comparison to previous approaches based on cloning and Sanger sequencing [9]. However, accurately determining the allelic profile of the original sample remains challenging. The most common approach to genotyping a multigene family starts with the design of a pair of primers targeting the genomic region of interest and then PCR amplification produces the library of DNA molecules to be sequenced. Such molecules should ideally come from every gene copy of the individual and their ratios should reflect those in the sample. If primers fail to amplify some of their intended targets, such targets will be missed in all subsequent analyses, and any amplification bias will distort the relative representation of targets in the analyzed sample. Moreover, the increase in sensitivity raises the need to discriminate contamination and systematic errors from real alleles [10].

In particular, Major Histocompatibility Complex (MHC) genes represent a functionally important multigene family that plays a key role in initiating the vertebrate immune response [11]. MHC genes are increasingly investigated in wild populations of non-model organisms by a high-throughput sequencing approach in order to understand the evolutionary significance of adaptive genomic variation in parasite and pathogen resistance [12–14]. Unfortunately, MHC locus-specific genotyping is usually troublesome because the gene family is prone to gene conversion, intergenic recombination, and high allelic variation. Most species–even those with whole genome drafts available–lack a detailed genomic characterization of the MHC region, complicating primer design, the assignment of alleles to loci and the estimation of the number of amplified gene copies [15]. Therefore, in non-model species MHC genetic variation is commonly characterized through the sequencing of exons coding for antigen-binding domains, using relatively conserved flanking sequences to simultaneously amplify multiple target genes by PCR. In many cases primers are directly adopted across studies or designed on the basis of sequence information available for related species.

The reliability of multi-gene studies depends critically on comprehensive and robust allelic profiles in the target sample, but this can be severely limited by PCR amplification biases [16], a problem that becomes more severe as the complexity of the target increases. The competitive nature of PCR in conjunction with possible target-primer mismatches, or differences in length, secondary structures, GC content and/or concentration (copy number) among targets [17] can result in amplification biases. Several strategies have been proposed to minimize amplification bias, including the use of as few PCR cycles as possible [18], chemically modified primers [19], and a two-step PCR setup that minimizes amplification bias by reducing the number of PCR cycles in the first step (i.e., annealing to the flanking region) and leaving most of them to the second PCR which uses primers perfectly matching the 5’ tail [20]. PCR replication with the same primer set has also been proposed as a method to control for stochastic amplification biases [21]. However, PCR replicates are of little use when the biases are intrinsic to specific primer-target combinations, like those caused by primer mismatches. While additional replicates will increase the chances of detecting some moderately amplified alleles, poorly amplified ones might be consistently missed across PCR replicates.

A second important issue in the application of PCR-based high-throughput approaches is the discrimination of sequencing errors and amplification artefacts from true alleles, which is exacerbated by amplification biases. Quality controls and data validation protocols have been specifically designed for MHC genotyping based on high-throughput sequencing (reviewed in [10]). Using different approaches, these validation protocols focus on reducing type I errors, i.e., not miscalling an artefact as a true allele. However, most of these protocols pay little or no attention to the minimization of type II errors, i.e., not calling true alleles despite being present in the sample. All current methods–except the ones by Sommer et al. [21] and Stutz & Bolnick [22]–assume that reads coming from true alleles are more abundant in the read pool than any artefactual sequence. If severe enough, amplification biases could lead to some true alleles being amplified at lower levels than artefacts or not being amplified at all, resulting in allelic-dropout [21,23,24]. Furthermore, the use of ultra-deep sequencing [10,24,25] cannot solve this problem when biases are quite severe, as increasing the coverage raises the probability of producing some reads from a poorly amplified allele but does not necessarily change its position in the coverage ranking.

To ensure the even amplification of all gene copies, primers are often designed with degenerate bases to account for all known variants in flanking sequences. Usually, the more complex the mixture of targets, the more degenerate the primers are designed–sometimes up to the point of producing side effects. If primers are highly-degenerate, many suboptimal primers specified by non-desired combinations of degenerate positions are synthesised, decreasing the effective concentration of the intended ones. The latter may be rapidly consumed in the PCR affecting amplification efficiencies, especially when they prime many loci. Furthermore, unwanted regions could co-amplify along with the region of interest.

To circumvent the issues associated with the use of highly-degenerate primers we propose two different approaches that may effectively characterize multigene families with gene copies differing in flanking primed sequences. In the first approach (hereafter pooled-PCRs strategy), different slightly-degenerate primer pairs are designed to preferentially amplify different subsets of paralogs that complement each other. They are used in separate PCRs whose products are later pooled for sequencing according to the number of expected loci targeted in each PCR. The second approach (hereafter pooled-primers strategy) consisted of a set of nondegenerate primers that were pooled following the same rationale as in the pooled-PCRs strategy (the primers matching more loci are more abundant in the primer mix) and a single PCR is performed.

**Fig 1:**
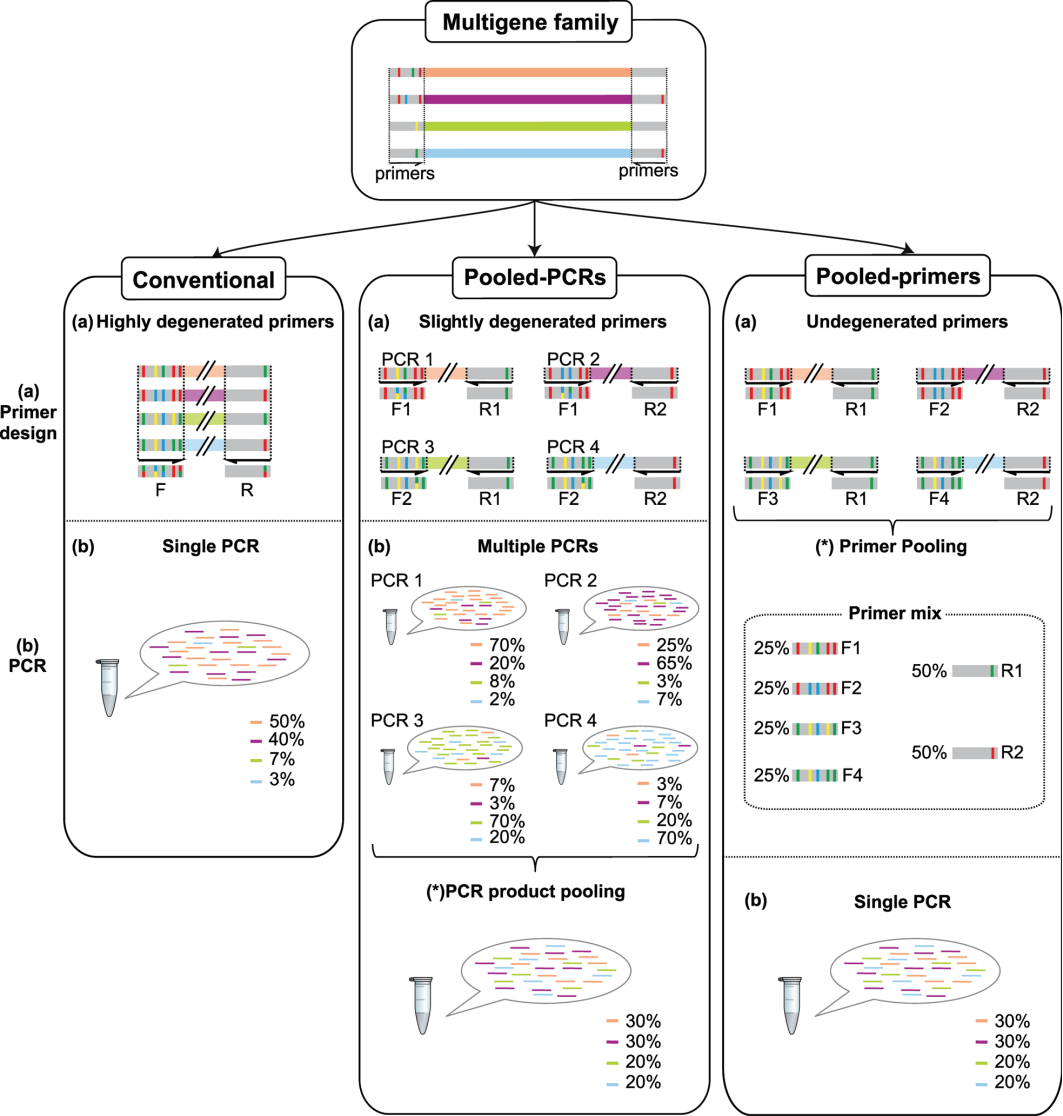
Diagram depicting the three alternative multigene family amplification strategies compared in this study. When amplifying multigene families, or other complex targets, some alleles may contain variants in the priming region, here reflected by colored bars. (a) Primer design: The conventional strategy tries to match all known variation by designing highly degenerate primers, but some variants might be missed because of unknown variation or in an attempt to avoid highly-degenerate primers. Degenerate nucleotides in the primers are represented by bars with more than one color. In the pooled-PCRs strategy, allele groups with similar priming regions are targeted separately with low-degeneracy primers and by taking into account the observed phase. In the pooled-primer approach, non-degenerate primers targeting each known flanking region are pooled according to the expected number of targeted alleles. (b) PCR amplification: The conventional and pooled-primers strategies amplify all alleles in a single PCR while in the pooled-PCRs strategy an independent PCR is performed for each primer pair. Both the conventional and pooled-PCRs approaches yield unbalanced libraries, but in the case of pooled-PCRs each library is biased toward a different set of alleles. Biases in the pooled-primers approach are minimized because all alleles are primed by perfectly matching primers at the right concentration. (c) PCR yield pooling: This step is exclusive to the pooled-PCRs approach and attempts to produce a final balanced sequencing library by pooling independent PCRs taking into account the number of perfectly targeted alleles. Note that this diagram is a sketch for illustrative purposes only and does not reflect the alleles, primers or libraries used in this study.

Here we evaluate whether the proposed pooled-PCRs and pooled-primers strategies result in more even amplification efficiencies and more reliable and complete genotypes when compared to the standard use of a single PCR with highly degenerate primers (hereafter conventional strategy) (Fig 1). If so, they would substantially improve the genotyping of multigene families as a result of a reduction in allelic-dropout rates. Hence, less coverage will be required per sample to achieve a complete profile, and it would also allow a more efficient discrimination of artefacts and errors. We tested and evaluated our different amplification strategies by characterizing the MHC class I genes of the Iberian lynx, a highly endangered species whose conservation strategies could benefit from an assessment of its MHC variation.

## Materials and Methods

Sampling procedures were licensed by the corresponding local Competent Authority to comply with Spanish legislation on the protection of animals used for scientific purposes.

### Primer design and amplification strategies

Primer design was based on a set of variants that included all MHC class I exon 2 alleles for species closely-related to Iberian lynx available in GenBank as of May of 2011 (i.e., all Felidae alleles. S2 File) and the annotated region of the cat [26]. We also used transcripts from two different lynxes annotated as MHC class I by the Iberian lynx genome project (unpublished data [27]). To be considered, any single nucleotide variant had to be present in: i) at least three variants of any felid species, ii) in two different felid species, iii) in any cat MHC class I classical locus or iv) in any Iberian lynx transcript. Primers were designed in approximately the same regions as the ones used in previous studies on MHC variation in the Felidae [28–30] (Table 1). More specifically, primers spanned bases 2 to 21 (forward) and 252 to 271 (reverse) of the human MHC class I exon 2 [31]. Primer design and primer pairing took into account the fact that single nucleotide variants in the same haplotype should not be separated into two different primers or primer pairs. DNAsp v5 [32] used to collapse the information available into unique sequences of the targeted regions and Primer3 [33] was used for primer design (Further detail in S1 File). Primers carried a universal 5’ extension to allow the adaptation of PCR products for 454 sequencing following the Universal Tailed Amplicon Sequencing design by Roche.

**Table 1:** PCR primers designed to target exon 2 of MHC class I genes in Iberian lynx.

					Degenerate		Uniq
Primer name	Primer short name	Sequence	Sense	Strategy	2 fold	3 fold	
Fel_MhcI_ex2_single_F	f	GCTCCCA**Y**TCC**Y**TGA**K**GTAT	Forward	Conventional	3	0	8
Fel_MhcI_ex2_F1	f1	GCTCCCACTCCCT**S**AGGTAT	Forward	Pooled-PCRs	1	0	2
Fel_MhcI_ex2_F2	f2	GCTCCCA**Y**TCCTTGA**K**GTAT	Forward	Pooled-PCRs	2	0	4
*Consensus Pooled-PCRs F*		GCTCCCA**Y**TCC**Y**T**S**A**K**GTAT	Forward	Pooled-PCRs	4	0	6
Fel_MhcI_ex2_Fa	fa	GCTCCCACTCCCTGAGGTAT	Forward	Pooled-primers	0	0	1
Fel_MhcI_ex2_Fb	fb	GCTCCCATTCCTTGATGTAT	Forward	Pooled-primers	0	0	1
Fel_hcI_ex2_Fc	fc	GCTCCCACTCCCTCAGGTAT	Forward	Pooled-primers	0	0	1
Fel_MhcI_ex2_Fd	fd	GCTCCCACTCCCTGCGGTAT	Forward	Pooled-primers	0	0	1
Fel_MhcI_ex2_Fe	fe	GCTCCCACTCCTTGAGGTAT	Forward	Pooled-primers	0	0	1
*Consensus Pooled-Primers F*		GCTCCCA**Y**TCC**Y**T**S**A**K**GTAT	Forward	Pooled-primers	4	0	5
Acju_Ex2MhcI_cF		GCTCCCACTCCCTGAGGTAT	Forward	Castro-Prieto et al., 2010	0	0	1
α1_F		CCACTCCCTGAGGTATTTCTACACC	Forward	Sachdev et al., 2005	0	0	1
Fel_MhcI_ex2_single_R	r	GG**MY**TCGCTCTGGTTGTAGT	Reverse	Conventional	2	0	4
Fel_MhcI_ex2_R1	r1	GG**MY**TCGCTCTGGTTGTAGT	Reverse	Pooled-PCRs	2	0	4
Fel_MhcI_ex2_R2	r2	GGAA**Y**CGCTCTGGTTGTAGT	Reverse	Pooled-PCRs	1	0	2
Fel_MhcI_ex2_R3	**r3**	**S**GAC**W**CGCT**Y**TG**R**TTGTAGT	Reverse	Pooled-PCRs	4	0	16
*Consensus Pooled-PCRs R*		**S**G**MHH**CGCT**Y**TG**R**TTGTAGT	Reverse	Pooled-PCRs	4	2	22
Fel_MhcI_ex2_Ra	ra	GGACTCGCTCTGGTTGTAGT	Reverse	Pooled-primers	0	0	1
Fel_MhcI_ex2_Rb	rb	GGCTTCGCTCTGGTTGTAGT	Reverse	Pooled-primers	0	0	1
Fel_MhcI_ex2_Rc	rc	GGACACGCTTTGATTGTAGT	Reverse	Pooled-primers	0	0	1
Fel_MhcI_ex2_Rd	rd	GGACTCGCTTTGGTTGTAGT	Reverse	Pooled-primers	0	0	1
Fel_MhcI_ex2_Re	re	GGAATCGCTCTGGTTGTAGT	Reverse	Pooled-primers	0	0	1
Fel_MhcI_ex2_Rf	rf	CGACTCGCTCTGGTTGTAGT	Reverse	Pooled-primers	0	0	1
Fel_MhcI_ex2_Rg	rg	GGAACCGCTCTGGTTGTAGT	Reverse	Pooled-primers	0	0	1
*Consensus Pooled-Primers R*		**S**G**MHH**CGCT**Y**TG**R**TTGTAGT	Reverse	Pooled-primers	4	2	7
Acju_Ex2MhcI_kR		GGA**K**TCGCTCTGGTTGTAGT	Reverse	Castro-Prieto et al., 2012	1	0	1
α1_Rb		GGACTCGCTCTGGTTGTAGTAGCG	Reverse	Sachdev et al., 2005	0	0	1

The sequences named as consensus represent the sum of all primers used in either the pooled-PCRs strategy or pooled-primers strategy and illustrates the encompassed variation. Variable bases are indicated by ambiguity codes in bold. For each primer set the number of bases with two-fold or three-fold degeneration is indicated (Degenerate 2-fold and 3-fold respectively), along with the number of unique sequences (uniq. i.e., the number of different non-degenerate sequences) the primer set includes. Other primers previously used in felids are included to show the greater flexibility of our approach.

For the pooled-PCRs strategy, we designed primer pairs with complementary specificity, so that all target sequences could be amplified by at least one pair in an independent PCR. We obtained two forward and three reverse low-degeneracy primers that were combined into four different primer pairs (S1 File). We performed independent PCRs with each primer pair and pooled them on the basis of the number of loci expected to be amplified in each PCR as inferred from the sequences used for primer design (i.e., adding more PCR product to the pool of those expected to target a higher number of alleles).

Regarding the pooled-primers strategy, we designed 5 forward and 7 reverse non-degenerate primers that were mixed prior to amplification following the same rationale as in pooled-PCRs approach: the more alleles a primer is expected to target the more concentrated it is in the pool (see Fig 1 and S1 File for further details).

To enable comparisons with standard high-throughput genotyping methods, we used the conventional strategy. To this end we designed a degenerate primer-pair aiming to include as many haplotypes as possible while keeping the base degeneration at reasonable levels. It must be noted that this pair is still less stringent than the ones previously used in felids [28–30] (Table 1), but it does not include all the variants included in the pooled-PCRs and pooled-primers approaches.

We did not use genomic information describing the expected loci to be amplified and transcriptomic information was not quantitatively useful as the assembler (Trinity [34]) produces many alternatively spliced transcripts and does not resolve transcripts of similar paralogs properly. Instead, we used as a proxy: i) the number of sequences targeted in out alignment -the higher the more prioritized- and ii) the range of covered species (or cat’s MHC described locus) -the broader the more prioritized (S2 File). With such information the resolution to adjust the pooling is rather low, so our pools consisted in about 50% of the primers/primer-pairs that targeted the haplotypes encompassing most of the sequences of the alignment and distributed the remaining 50% among the rest (giving double % to some of them) (S1 File).

### Amplification and sequencing of MHC class I loci

Genomic DNA was extracted from blood or muscle of 18 different Iberian lynxes using standard phenol-chloroform methods [35] and then the three amplification strategies were tested on the same extract for each individual. We used the Universal Tailed Amplicon Sequencing design by Roche consisting of a two-round PCR approach in which the first round amplifies the target locus using primers with a universal 5’ extension and the second adds 454 sequencing adapters and an individual tag to the amplicons generated in the first PCR. Amplicons were tagged so that sequences could be sorted by sample and strategy (i.e. the four amplicons per individual generated using pooled-PCRs strategy were given the same tag). Artefact formation during PCR was minimized by using the Phusion^®^ High-Fidelity PCR Kit by Roche, which reduces nucleotide misincorporation [36], and by implementing long extension times and no final extension step, which should prevent chimera formation. PCRs were run in a final volume of 10 μl following manufacturer indications. Cycling conditions were the same for all PCRs: an initial denaturation at 98° for 30 sec and 25 cycles of 98°C for 10 sec, 57°C for 30 sec and 72°C for 2 min. PCR products were pooled and sequenced on a 454 GS Junior system.

### High-throughput data processing, alleles validation protocol and amplification efficiencies

Sequences were quality filtered, sorted by strategy and individual, and assigned to alleles or artefacts following Sommer et al. [21]. The only modification introduced to the latter strategy involved the non-systematic replication of samples. As the species genetic diversity is very low [37], we expected every allele to be found in more than one individual. Nevertheless, 11% of the samples were replicated as a quality control. Briefly, the methodology scored as artefacts all the chimeras (refer to Sommer et al. [21] for details), singletons (i.e. variants appearing only once in a PCR), and more frequent (n>1) variants containing two or less differences to a higher frequency variant within the same PCR and not present in an independent PCR. Variants with more than 2 differences to all higher frequency variants within the same PCR but not present in any other independent PCR were considered as “unclassified” and were further manually checked. Later, it scored as alleles the most frequent variant in each independent PCR as well as variants that were already scored as alleles in other individuals and present a higher frequency than all artefacts within the same PCR. When such variants presented a lower frequency than artefacts, they were manually checked and still considered as alleles if such artefacts were explained as artificial chimeras from high frequency alleles (or in special cases, a verified biased error in the sequencing technology).

We chose the validation protocol of Sommer et al. [21] because it assumes different amplification efficiencies for different alleles and does not assume that reads representing true alleles are more abundant in the read pool than any artefact. These two conditions are important when primers are not expected to amplify alleles with the same efficiency. Finally, we calculated the standardized amplification efficiencies for each allele in the three amplification strategies using the R codes provided in Sommer et al. [21] and taking the least amplified allele as reference.

### Allelic profile reliability and completeness

To test how efficient the strategies were in capturing genomic variation at MHC class I loci we compared the allelic profile obtained with each strategy for each sample to the profile inferred from the combination of the sequencing data of the three approaches. Note that this represents only the most complete profile given the available data and thus was considered the best approximation to the true genotype of the individual. We then compared the profile obtained with each strategy for each sample to the “complete” profile of the sample. The probability of detection for each allele was calculated for each strategy as the number of individuals in which the allele was scored using the strategy divided by the number of individuals for which the allele was scored in the “complete” genotype.

For each strategy and sample we calculated the number of missed alleles, i.e., the alleles found to be present in the individual but not detected by this strategy, and profile completeness, i.e., the percentage of the alleles observed with respect to the number of alleles present. Moreover, we calculated the percentage of the reads that each allele copy attained with respect to the total number of reads corresponding to alleles (i.e., %reads allele_i_ = reads allele_i_ / (Σreads alleles x copy number_i_)), taking copy number as 1 or 2 for heterozygote and homozygote loci respectively.

### Evaluation of the impact of coverage depth on allele detection through simulations

To directly test whether increasing the coverage compensates for less balanced amplification efficiencies, we tested different coverage scenarios for each strategy. Empirically, all amplicons were assayed in the range of ultra-deep sequencing (i.e., hundreds to thousands of reads per amplicon), but we simulated lower coverages by bootstrapping 100 times the whole set of reads obtained from each amplicon, sampling from 0 to 4500 reads in incremental steps of 10 reads, and subsequently scoring alleles using a Perl script. We then plotted the allelic profile completeness of those simulations as accumulation curves using R[38].

### Statistical analysis

We tested whether the pooled-PCRs and pooled-primers strategies produced more even amplification efficiencies (i.e., variance is lower) than the conventional approach by normalizing the distributions using log transformation and applying the parametric F test to compare two variances (one-tailed). To test whether the “alleles more frequent than artefacts” assumption was met in our experiment and to assess to what extent validation protocols requiring that assumption to be met could have biased our results, we calculated the percentage of times that each allele was observed at a coverage lower than the most common artefact in an amplicon by interrogating our SQL databases with a Python script. All statistical tests were implemented in R [39].

### Flanking sequences information

To assess whether mismatches between primers and the corresponding genomic sequences were the major cause of unbalanced amplification efficiencies we retrieved information about the genomic regions flanking each allele from lynx genome scaffolds matching the observed alleles using Megablast searches (default parameters as defined in Geneious R7[40]), and from genes annotated as MHC class I genes in the Iberian lynx genome draft[27]. We compared the set of alleles found in the genome draft with the ones detected by amplicon typing this same individual, and evaluated to what extent differences in amplification efficiencies between strategies could be explained by primer-template mismatches.

## Results

### Data quality control and allele validation

We obtained a total of 169,485 reads corresponding to MHC class I exon 2 amplicons from four different 454 Jr. runs, which were shared with other projects. All 18 samples reached a coverage over 750 reads in all three strategies. Using the conventional approach the average coverage per sample was 1,325 (range: 757–5,420), in the pooled-PCR strategy 2,016 (range: 926–3,648) and in the pooled-primers set-up 6,075 (range: 1,631–16,729).

We validated 13 putative MHC class I alleles in total. While all 13 putative alleles were scored by the pooled-PCRs and pooled-primers strategies, the conventional approach failed to score one of them. This missing allele (Lypa-MHCI*6) was the most poorly amplified in the latter set-up and was in fact detected in one sample, but it was not scored due to lack of replication and thus called an artefact in the conventional approach. Nevertheless, we kept it in the analysis for comparison purposes (Tables 2 and 3).

**Table 2:** Relationships between average allele coverage, probability of detection, and the number of matching PCRs.

**Conventional**																
Allele name	% reads	Probability of detection	PCR match	Pool match	f-r 100%											
Lypa-MHCI*2	16.36	1	1	1	0(0/0)											
Lypa-MHCI*4	7.78	0.94	0	0	1(1/0)											
Lypa-MHCI*5	6.85	0.94	0	0	1(1/0)											
Lypa-MHCI*11	3.23	0.86	1	1	0(0/0)											
Lypa-MHCI*8	0.80	0.44	1	1	0(0/0)											
Lypa-MHCI*7	0.36	0.17	0	0	1(0/1)											
Lypa-MHCI*9	0.24	0.44	0	0	1(1/0)											
Lypa-MHCI*6	0.13	0.06	0	0	3(0/3)											
Lypa-MHCI*14	0.00	0.00	0	0	2(1/1)											
**Pooled-PCRs**																
Allele name	% reads	Probability of detection	PCR match	Pool match	f1-r1 50%	f2-r1 16.6%	f1-r2 16.6%	f1-r3 16.6%								
Lypa-MHCI*4	15.97	1	2	0.67	0(0/0)	2(2/0)	1(0/1)	0(0/0)								
Lypa-MHCI*9	4.86	1	2	0.67	0(0/0)	2(2/0)	1(0/1)	0(0/0)								
Lypa-MHCI*11	3.90	1	2	0.67	0(0/0)	1(1/0)	1(0/1)	0(0/0)								
Lypa-MHCI*8	3.47	1	2	0.67	0(0/0)	1(1/0)	1(0/1)	0(0/0)								
Lypa-MHCI*2	2.34	0.83	1	0.17	3(3/0)	0(0/0)	5(3/2)	5(3/2)								
Lypa-MHCI*5	1.39	1	0	0.00	1(1/0)	2(2/0)	2(1/1)	1(1/0)								
Lypa-MHCI*6	1.06	0.94	1	0.17	3(0/3)	4(1/3)	4(0/4)	0(0/0)								
Lypa-MHCI*7	0.84	0.94	1	0.17	1(0/1)	2(1/1)	2(0/2)	0(0/0)								
Lypa-MHCI*14	0.00	0.00	0	0.00	4(3/1)	3(2/1)	5(3/2)	4(3/1)								
**Pooled-primers**																
Allele name	% reads	Probability of detection	PCR match	Pool match	fa 45.5%	fb 18.2%	fc 18.2%	fd 9.1%	fe 9.1%	ra 41.7%	rb 16.7%	rc 8.3%	rd 8.3%	re 8.3%	rf 8.3%	rg 8.3%
Lypa-MHCI*4	6.00	1	1	1	3	4	0	2	2	0	2	3	1	1	1	2
Lypa-MHCI*8	5.49	1	1	1	0	3	1	1	1	0	2	3	1	1	1	2
Lypa-MHCI*7	4.31	1	1	1	0	3	1	1	1	1	3	2	0	2	2	3
Lypa-MHCI*11	3.83	1	1	1	0	3	1	1	1	0	2	3	1	1	1	2
Lypa-MHCI*5	2.72	1	1	1	1	4	2	0	2	0	2	3	1	1	1	2
Lypa-MHCI*9	1.60	1	1	1	1	4	0	2	2	0	2	3	1	1	1	2
Lypa-MHCI*6	1.51	1	1	1	0	3	1	1	1	3	5	0	2	4	4	4
Lypa-MHCI*2	1.41	1	1	1	1	0	4	4	2	2	0	5	3	2	3	3
Lypa-MHCI*14	0.00	0	0	0	3	4	4	4	2	1	3	4	2	2	2	3

For each allele for which genomic data of the flanking region is available, the average percentage of reads corresponding to each allele copy (%reads) is shown, as well as the number of independent PCRs perfectly matching the allele’s priming sequences (PCR-match), the number of perfectly matching PCRs weighted by their proportion in the sequencing pool (if the allele is perfectly matched by primers in all four PCRs it would be 1 (Pool match)). The following columns depict the primers used in each PCR followed by the proportion of their corresponding product in the final sequencing pool and the number of total mismatching bases for each allele (mismatches primer F / mismatches primer R); see S2 Table for further details. In the pooled-PCRs strategy primers used in separate PCRs complement each other, so that most alleles are perfectly matched in at least one independent PCR. Similarly, in the case of pooled-primers all alleles are perfectly matched by at least one of the non-degenerate primers used. Alleles not present in the genome assembly are not shown.

**Table 3:** Comparison of the general performance of the three approaches and expected values.

	Alleles detected		
Genotyping strategy	Population level	Individual level	Alleles lost
Conventional	12*	7.94 ± 1.95	3.83 ± 1.68
Pooled-PCRs	13	11.33 ± 1.38	0.44 ± 1.04
Pooled-primers	13	11.72 ± 0.89	0.05 ± 0.24
Expectation	13	11.78 ± 0.88	-

The pooled-PCRs and pooled-primers strategies detected all alleles at a population level whereas the conventional approach missed one (* even though Lypa-MHCI-06 is seen in one amplicon it was not scored as an allele due to lack of replication). The pooled-PCRs and pooled-primers strategies yielded more accurate and complete allelic profiles, as they consistently detected most of the alleles present in the individuals. The expected values reflect the inferred complete profiles for each assayed individual.

### Allele detection and amplification efficiencies

**Fig 2:**
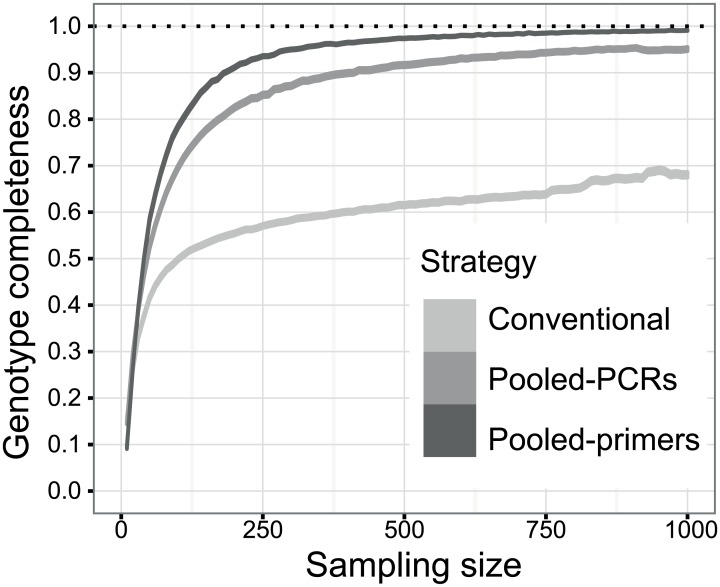
Allelic profile completeness obtained with increasing coverage. The same set of individuals was assayed with the three strategies and the reads obtained for each amplicon were bootstrapped to simulate lower coverages (increasing steps of 10 reads, 100 iterations). Profile completeness is defined as the proportion of the alleles in the individual’s inferred profile (from the pooling of all available data) that were scored in each iteration. Both the average value and its confidence interval (95%) are represented. Note that increasing the coverage does not compensate for highly biased amplification efficiencies (see S2 Fig for larger sampling sizes).

The number of validated alleles per individual–taking into account the total evidence–ranged from 11 to 13, indicating that some individuals showed all alleles detected and that the minimum possible number of loci amplified was seven. The average number of alleles detected per individual in the pooled-PCRs strategy was 11.33 ± 1.38 (range 9–13), and 11.72 ± 0.89 (range 9–13) in the pooled-primers one. Both were higher than the average for the conventional approach, which was only 7.94 ± 1.95 (range 5–12). This yielded a percentage of complete profiles of 96.28 ± 8.64, 99.50 ± 2.12 and 66.67 ± 14.21 for the pooled-PCRs, pooled-primers and conventional strategies, respectively (Table 3, Fig 2).

**Fig 3:**
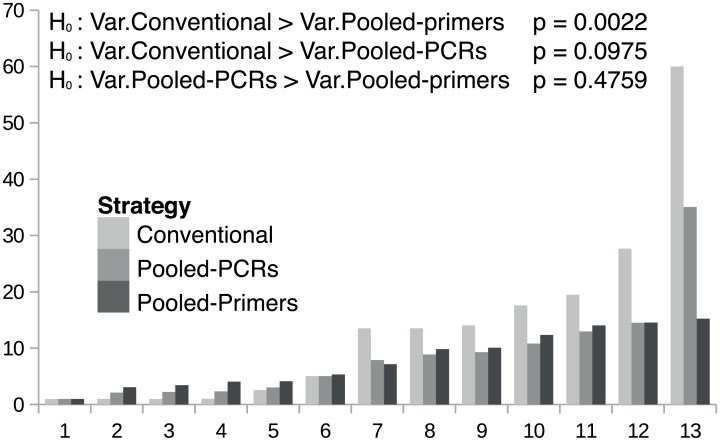
Standardized amplification efficiencies for each allele obtained with the three amplification strategies. For easier comparison alleles are ranked from one (the lowest) to 13 (the highest) within each strategy, and thus numbers do not identify specific alleles. The range of standardized amplification efficiencies is approximately two times and four times larger using the conventional strategy than the pooled-PCRs and pooled-primers approaches, respectively.

The pooled-primers strategy yielded significantly more even amplification efficiencies than the conventional one (F test, p<0.05), whereas for the pooled-PCRs set-up the difference to the conventional approach was not significant (F test, p = 0.0975) (Fig 3). The standardized amplification efficiency variance in the conventional strategy was more than three times the variance obtained by the pooled-PCRs approach, and ten times that of the pooled-primers strategy. While in the conventional approach the worst amplified allele, measured as the percentage of reads corresponding to each allele copy in the genome, was about 130 times less covered than the best amplified, this figure dropped to 30 in the pooled-PCRs strategy and to 13 in the pooled-primers one (S1 Table).

Alleles with lower probabilities of detection were those with lower amplification efficiencies (Table 2 and S1 Table), especially for the conventional strategy in which the differences in efficiencies were more severe. In the pooled-PCRs strategy, the higher number of independent PCRs perfectly targeting an allele resulted in a higher amplification efficiency and, therefore, in an increased probability of detection (Table 2). Moreover, the worst five amplified alleles attained lower frequencies than the most common artefact in half of the amplicons using the conventional strategy, whereas this only occurred with one allele in the pooled-primers approach and not at all in the pooled-PCRs strategy due to their more even amplification efficiencies (see S1 Fig for further details). Hence, for the conventional and the pooled-primers approaches, the “alleles more frequent than artefacts” assumption was not met. The rank of alleles by amplification efficiency was not maintained between approaches, indicating that changing the primers used affected amplification efficiency irrespective of other intrinsic characteristics of the allele.

### Ultra-deep coverage effect

The plots of allele accumulation by increasing coverage through simulations (Fig 2) illustrate a clear pattern: within any strategy increasing the coverage improves the profile completeness until it reaches a plateau, after which a further increase hardly improves the resulting profiles (see a wider range of coverage in S2 Fig). The key difference between the three strategies lies in the coverage required in order to reach the plateau and the level of completeness this saturation point provides. While the pooled-PCRs and pooled-primers strategies surpass 90% of called alleles with as little as 420 and 190 reads respectively, the conventional approach never reaches that point. Indeed, at best the conventional approach achieved 73% of completeness with 4,500 reads coverage (the highest value simulated).

### Primer mismatch analyses

The Megablast search against the Iberian lynx genome assembly produced the same 10 highly significant full-length hits, irrespective of the allele used as query. Nine corresponded to alleles scored by this study in the genome-sequenced individual (one of them was not assembled full-length), and were annotated as MHC class I. The remaining hit had not been previously detected through sequence-based typing nor annotated as MHC class I, despite showing high similarity to other alleles. The assembly missed two alleles scored in the genome-sequenced individual, which might correspond to heterozygous or unassembled loci.

The independent PCRs performed in the pooled-PCRs strategy targeted different subsets of alleles as intended, with nearly all alleles perfectly matching primers in one or more PCRs, the only exceptions were one allele with a mismatch in one of the flanking regions (Lypa-MHCI*5) and another with one mismatch at each flank (Lypa-MHCI*14). One of the independent PCRs (Fel_MhcI_ex2_F1-Fel_MhcI_ex2_R2) did not match perfectly to any of the alleles found in the genome draft and seems unnecessary in view of the genomic information. A similar situation was found using the pooled-primers strategy, in which all alleles perfectly matched two of the non-degenerate primer used. In the conventional strategy only 4 alleles perfectly matched the primers, four exhibited a mismatch in one of the flanks, one presented two mismatches in the reverse flanking region, and one allele showed one mismatch at each flank. None of the mismatches were located at the most critical bases near the 3’ extreme of the primer (S2 Table).

The difference in amplification efficiencies among alleles and between strategies (Table 2) can be partly explained by primer mismatches and the number of independent PCRs amplifying each allele. Alleles that do not perfectly match any of the primers included in the conventional strategy are amplified less efficiently or missed altogether. Interestingly, slight primer changes translated into huge amplification efficiency shifts. For example, allele Lypa-MHCI*14 which is present in the genome assembly but is not scored by any of the strategies used shows one single primer mismatch in each flanking region. Notably, the outcome depends on the competition context in which the alleles are amplified, i.e., it is the efficiency relative to other competing alleles that is important. The allele ranked 8^th^ in the pooled-PCRs strategy performs the best in the pooled-primers approach because it takes great advantage of a single mismatch that slightly discriminates against the rest.

## Discussion

Here we evaluated two novel approaches for characterizing multigene families by PCR and high-throughput sequencing using MHC class I multi-locus genotyping as an example. Our results show that the traditional use of a single highly degenerate primer pair resulted in unreliable and incomplete profiles due to amplification biases among alleles. While an increased sequence depth (i.e., coverage) improved the results, it could not fully compensate for the unbalanced amplification of the alleles. In contrast, the use of several independent PCRs using moderately degenerate primer pairs with complementary specificity or a single PCR using a pool of complementary and non-degenerate primers can homogenize the probability of detection of alleles, increasing profile reliability even at much lower coverages.

Our study identified amplification biases derived from large differences in amplification efficiencies among targets as a major limitation for the characterization of multigene families through the high-throughput sequencing of PCR amplicons. Such biases limit the reliability of individual genotyping of complex gene families like MHC, KIR, TLR, etc. The low amplification efficiencies obtained for some alleles were in large part attributable to primer mismatches, i.e., less efficiently amplified or missed alleles were flanked by sequences not completely matching any of the primers or matching them in fewer of the four independent PCRs. Primer mismatches are known to affect amplification efficiencies and the magnitude of the effect depends on many factors: the mismatch nature (transition or transversion), primer length, its relative position within the primer, and its neighbouring sequence [16,41–47]. However, in our case mismatches were always single mismatches at least 1 base away from the 3’ end, the most critical for amplification efficiency [47]. Such minor-effect mismatches would have not prevented the amplification if occurring in isolation, but caused allelic-dropout when amplified in competition with other perfectly matching sequences. Moreover, we found that amplification efficiency shifts are difficult to predict and context-dependent. A great efficiency increase of allele Lypa-MHCI*2 in the conventional approach can be attributed to a change in the amplification context, showing that when primers favour or disfavour a certain allele, it affects not only its own amplification efficiency, but also that of all the others. Although we were unable to test this interpretation directly due to a low number of similar genotypes, this indicates that allele amplification efficiencies may be sample dependent. Non-random errors might confound the amplification efficiency in high-throughput sequencing assays when artefactual read counts are not added to the read counts of their parental variants. Alleles bearing sequence motifs prone to cause sequencing errors may produce a higher number of artefactual reads, which are removed during processing. This could distort the final number of reads retrieved per allele, mimicking a less efficient amplification. Some validation protocols [24,48] address this issue. Even though our protocol [21] does not take this concern into consideration, our validated alleles bear a similar number of long homopolymer runs (the most frequent artefact in 454 runs) and no correlation between the number of long homopolymer runs present in a certain allele and its amplification efficiency was observed (data not shown). Therefore we think our results are not affected by such an issue.

Our results highlight the paramount importance of primer design in this kind of study. Given that new data becomes available at increasing rates, the redesign of primers with up-to-date information should be the standard practice, in contrast with the common practice of borrowing them from previous studies. While it is true that most non-model species lack fine-scale genomic information, especially for the MHC region and other multigene families that are difficult to assemble, an exhaustive review of the information available for related species should be of great help. In our case, we were able to use transcriptomic data obtained for the species, but a very similar set could have been designed solely taking into account the information for other felids that was publicly available. Obviously, in complete absence of information, even from related species, no primers can be designed. However, for those species lacking genomic/transcriptomic resources -but with information on related species- the design can rely on positions conserved across taxa in the 3’ most bases and the targeting all observed variants with either the pooled-PCRs or the pooled-primers approach. Pooling could be performed according to the criteria given (i.e, number of sequences targeted and number of observations across species), a small sequencing test containing several individuals encompassing the study genetic structure (if known) run, and primer pooling readjusted based on these preliminary results. As primers are sequenced along with the inserts, the information of which primer is preferentially amplifying each allele can be used to tweak the pooling design ad hoc. To do so, the primer-region sequenced along with each allele can be extracted and represented as a LOGO [49].

Most MHC studies aim to genotype all functionally relevant gene copies in the species genome. To our knowledge, the minimum number of targeted loci commonly described for MHC class I in other felid studies ranges from three to five [28–30,50]. Our approach raised it to seven; however, population genetic analyses suggest that there are actually 11, many of them being monomorphic (Marmesat et al., in preparation). Given that the only felid with an extensively characterized MHC region [26]–the domestic cat–harbors 19 MHC class I loci, and that only one of the alleles found in the lynx genome was not scored, we think that both pooled-PCRs and pooled-primers strategies succeeded in screening a substantial fraction of MHC class I loci, and certainly more than any previous genotyping approach used in wild felids.

Most importantly, we showed that amplification biases cannot be fully compensated for by increased coverage. Although we did confirm the expected positive effect of increasing sequencing depth on profile completeness (Fig 2) [10,24,25] less efficiently amplified alleles failed to be detected even at ultra-deep coverage (see conventional strategy, Table 3). Furthermore, some poorly amplified alleles consistently attained lower coverages than the most common artefact, especially in the case of the conventional approach (S1 Fig). Thus, we think that the “true alleles should be observed at greater depths than artefacts” assumption [10,23] cannot be embraced by default, as already recognized in some studies (e.g., [21,22,51]). We highlight the need for improved and more sophisticated validation protocols that take explicitly amplification biases into account. Missed alleles due to amplification biases may be a pervasive problem in MHC genotyping. In our study of Iberian lynx MHC diversity it could have misled inferences at a population and individual level–as it yielded a lower total number of alleles present in the population as well as incomplete individual profiles and biased allele frequencies. This would have severely affected conclusions based on these data regarding, for example, the level of MHC diversity or the impact of individual MHC genotypes on fitness. It remains to be seen to what extent the likely incomplete or inconsistent genotyping of MHC in non-model organisms is hampering the understanding of the evolutionary forces acting on these genes or the detection of genotype-fitness correlations in natural populations.

## Conclusions

Exhaustive and reliable profiling of multigene families remains a challenging task for those working with non-model organisms despite the increase of sequencing power and other technical advantages brought about by high-throughput sequencing. Our study clearly illustrates how conventional standard methods still do not accurately genotype all targeted gene copies when there are many of these and variability in flanking regions exists. Ultra-deep sequencing mitigates but does not solve the problem as one important limitation arises prior to the sequencing step, namely the biased representation of target sequences in the sequencing library. While amplification biases cannot be completely eliminated when preparing these libraries through PCR, we have empirically shown how the use of several complementary primer sets in independent PCRs or, better still, a single PCR with a pool of non-degenerate primers at carefully adjusted concentrations can substantially ameliorate this problem.

The use of multiple PCRs with primer sets of complementary specificity (pooled-PCRs strategy), or a single PCR with a mix of non-degenerate primers at concentrations adjusted for expected template abundance (pooled-primers strategy), greatly improved allele detection probabilities, and thus the methodology reliability. Alleles that were missed using the conventional method because of their low relative amplification efficiency could however be consistently genotyped with the pooled-PCRs and the pooled-primers approaches, even with significantly lower sequencing effort (Fig 2). The improved result must be attributed to the more even amplification efficiencies brought about by these strategies. The strategy behind the pooled-PCRs approach aims at reducing competition among targets by doing separate PCRs targeting different sets of templates. A similar rationale is applied when species-specific primers are used to complement the generalist primers in metabarcoding [52], or when different primer pairs are used to characterize MHCIIB variation in birds [53]. Such a strategy involves, however, additional experimental steps with subsequent extra costs in terms of reagents and labour, which may dissuade potential users–especially in more complex contexts where many independent PCRs may be required. The benefit in terms of accuracy and sensitivity might well compensate for the extra cost in many situations, but this must be carefully evaluated for every scenario. Even though we expected the pooled-PCRs strategy to be the most efficient and sensitive approach, this was not the case. This could have been caused by the unavoidable inaccuracies when quantifying and pooling independent PCR products, by the problems associated with the use of degenerate primers [20] or by the fact that primer pairs are designed according to known haplotypes which might prevent the amplification of alleles generated via recombination. The use of a single PCR with a mix of non-degenerate primers at concentrations adjusted to the expected template copy number (pooled-primers) yielded optimal results with little extra cost (i.e., the extra primer synthesis) over the standard single PCR with degenerate primers, and should thus be the strategy of choice in most situations.

## Supporting Information

S1 Fig Rank of alleles relative to the most frequent artefact. Rank of alleles relative to the most frequent artefact. Unique sequences within the amplicon are ranked by number of reads and the rank of the most frequent artefact is set at zero, so a positive value means that the alleles attained higher coverage than any artefact and vice versa. Note that alleles with low efficiency often reach lower coverage than the most common artefact, especially in the conventional strategy were the amplification efficiencies are more unbalanced.
(DOC)

S2 Fig Allelic profile completeness obtained in relation to increasing coverage. Allelic profile completeness obtained in relation to increasing coverage. The same set of individuals was assayed with the three strategies and the reads obtained for each amplicon were bootstrapped to simulate lower coverages (increasing steps of 10 reads, 100 iterations). Profile completeness is defined as the proportion of the alleles in the individual’s inferred profile (from the pooling of all available data) that were scored in each iteration. Both the average value and its confidence intervals (0.95%) are represented. Note that increasing the coverage does not compensate for highly biased amplification efficiencies.
(DOC)

S1 File Primer design summary. Contains: i) Genebank identifiers for sequences used in primer design, ii) Haplotypes present in the region considered for the forward and reverse primer design, iii) Detailed description of the primer and pooling design: First (1.a and 1.a), all haplotypes present on the regions evaluated for primer design are evaluated to be considered in primer design if they fulfill our criteria. Second (2.a and 2.b), considered haplotypes are targeted using the three amplification strategies. Last (3), for the pooled-PCRs strategy the pooling is designed.
(XLS)

S2 File Global alignment fasta file. MHC class I sequences alignment used for primer design. Sequences come from GenBank and the Iberian lynx transcriptomic data available at the moment.
(FASTA)

S1 Table Allele's coverage and probability of detection per amplification strategy. Allele's coverage (measured as the average percentage of reads corresponding to each allele copy, %reads) and probability of detection per amplification strategy. Alleles are ranked in descending %reads order.
(XLS)

S2 Table Number, nature and position of primer mismatches per allele and strategy. Number, nature and position of primer mismatches per allele in each strategy. For each allele for which we have genomic data about the flanking region we show their alignments with each primer-pair assayed in the pooled-PCRs strategy in the first four columns. Dots indicate identity to the top sequence, the backslash separates forward and reverse primers. Note that reverse primer is not reversed complemented so the closer to the backslash the closer to the target of amplification. The last four columns depict the primers used in each PCR followed by the proportion of their corresponding product in the final sequencing pool (in brackets) and the corresponding number of total mismatching bases for each allele (mismatches primer F / mismatches primer R). Degree of mismatch is additionally coded by different grades of shade (the darker the better matching). In the pooled-PCRs strategy primers used in separate PCRs complement each other so that most alleles are perfectly matched in at least one independent PCR. Alleles not present in the genome-assembly not shown.
(XLS)

S3 Table Final allelic profiles per approach and their comparison. Final allelic profiles per approach along with their union and intersections, and statistics about the number of scored and missed alleles and profile completeness.
(XLS)
