# Supplementary material for: PCR Strategies for Complete Allele Calling in Multigene Families Using High-Throughput Sequencing Approaches
Source: PLoS One. 2016 Jun 13;11(6):e0157402. doi: 10.1371/journal.pone.0157402 (PMC4905633; doi:10.1371/journal.pone.0157402)
Supplement: S1 Fig — Rank of alleles relative to the most frequent artefact. Unique sequences within the amplicon are ranked by number of reads and the rank of the most frequent artefact is set at zero, so a positive value means that the alleles attained higher coverage than any artefact and vice versa. Note that alleles with low efficiency often reach lower coverage than the most common artefact, especially in the conventional strategy were the amplification efficiencies are more unbalanced. (DOC) [file pone.0157402.s001.doc]

**Supplementary Figure 1**

Rank of alleles relative to the most frequent artefact. Unique sequences within the amplicon are ranked by number of reads and the rank of the most frequent artefact is set at zero, so a positive value means that the alleles attained higher coverage than any artefact and *vice versa*. Note that alleles with low efficiency often reach lower coverage than the most common artefact, especially in the conventional strategy were the amplification efficiencies are more unbalanced.
