# Supplementary material for: PCR Strategies for Complete Allele Calling in Multigene Families Using High-Throughput Sequencing Approaches
Source: PLoS One. 2016 Jun 13;11(6):e0157402. doi: 10.1371/journal.pone.0157402 (PMC4905633; doi:10.1371/journal.pone.0157402)
Supplement: S2 Fig — Allelic profile completeness obtained in relation to increasing coverage. The same set of individuals was assayed with the three strategies and the reads obtained for each amplicon were bootstrapped to simulate lower coverages (increasing steps of 10 reads, 100 iterations). Profile completeness is defined as the proportion of the alleles in the individual’s inferred profile (from the pooling of all available data) that were scored in each iteration. Both the average value and its confidence intervals (0.95%) are represented. Note that increasing the coverage does not compensate for highly biased amplification efficiencies. (DOC) [file pone.0157402.s002.doc]

**Supplementary Figure 2**

Allelic profile completeness obtained in relation to increasing coverage. The same set of individuals was assayed with the three strategies and the reads obtained for each amplicon were bootstrapped to simulate lower coverages (increasing steps of 10 reads, 100 iterations). Profile completeness is defined as the proportion of the alleles in the individual’s inferred profile (from the pooling of all available data) that were scored in each iteration. Both the average value and its confidence intervals (0.95%) are represented. Note that increasing the coverage does not compensate for highly biased amplification efficiencies.
